# Comprehensive genetic analyses of primary adrenal failure without enzymatic defects

**DOI:** 10.1186/1687-9856-2013-S1-P109

**Published:** 2013-10-03

**Authors:** Naoko Amano, Mie Hayashi, Satoshi Narumi, Koji Muroya, Rika Kizu, Hiroshi Mochizuki, Yuko Taniguchi, Hiroki Matsuura, Keiko Homma, Tomonobu Hasegawa

**Affiliations:** 1 Keio University School of Medicine, Tokyo, Japan; 2 Ohtsuka Metropolitan Hospital, Tokyo, Japan; 3 Kanagawa Children’s Medical Center, Kanagawa, Japan; 4 Yokosuka Kyosai Hospital, Kanagawa, Japan; 5 Saitama Children’s Medical Center, Saitama, Japan; 6 International University of Health and Welfare Hospital, Tochigi, Japan; 7 Shinshu University School of Medicine, Nagano, Japan; 8 Keio University Hospital, Tokyo, Japan

## 

Our objective is to estimate frequencies of mutations in *STAR*, *CYP11A1*, *NR0B1*, *NR5A1*, *MC2R*, and *MRAP* in a cohort of Japanese patients with primary adrenal failure without enzymatic defects. Twenty-one patients were included, who were diagnosed as having primary adrenal failure without enzymatic defect, namely 21-hydroxylase deficiency, 3βHSD deficiency, 11β-hydroxylase deficiency, and P450 oxidoreductase deficiency. Sixteen patients presented with primary adrenal failure before the age of 2 years. Fourteen patients had apparent mineralocorticoid deficiency. Fourteen patients were 46, XY and 7 patients 46, XX. Three had 46, XY disorders of sex development. Mutation analyses of *STAR*, *NR0B1*, *NR5A1*, *MC2R*, and *MRAP* were done by PCR-based sequencing and next generation sequencing. In case of no amplification of *NR0B1* by PCR, we performed oligonucleotide array CGH. We descried clinical findings in each patients and determined possible genotype-phenotype correlation. Five patients were diagnosed as having DAX-1 deficiency. *NR0B1* mutations were found hemizygously in 3 patients (c.116delG, c.846_865del, and p.Q283X). *NR0B1* deletions were found in 2 patients (400kb deletion including *NR0B1* and 2.4kb deletion of exon 1). Four patients presented with primary adrenal failure in newborn, and the other patient presented at the age of 6 years. *STAR* mutations were found in 3 patients. One patient was 46, XY, and 2 patients were 46, XX. One patient, who presented with primary adrenal failure in newborn, had c.712delA/p.Q258X. Two patients, who presented at preschool age, had p.Q258X/p.R272C and p.Q258X/p.R188H. No mutations were found in *CYP11A1*, *NR5A1*, *MC2R*, and *MRAP*. In conclusion, *NR0B1* mutations and deletions are relatively common in 46, XY normal male phenotype patients (5/11). *STAR* mutations might be found in cases, being older than 2 years of age. 3. *CYP11A1*, *NR5A1*, *MC2R*, and *MRAP* mutations are rare.

